# Recurrent Urinary Tract Infections in a Patient with Diffuse Large B-Cell Lymphoma and Severe COVID-19: A Single Case of Suspected Immunosuppression Where Antibacterial Therapy Was Not Enough

**DOI:** 10.3390/antibiotics15010048

**Published:** 2026-01-03

**Authors:** Paula Irina Barata, Liana Maria Chicea, Irena Nedelea, Carmen Nicoleta Strauti, Diana Deleanu, Maria Daniela Moț, Coralia Cotoraci, Cristian Oancea

**Affiliations:** 1 Department of Physiology, Faculty of Medicine, “Vasile Goldis” Western University of Arad, Blvd. Revolutiei, No. 96, 310025 Arad, Romania; barata.paula@uvvg.ro; 2 Department II Medical Clinic, “Victor Papilian” Faculty of Medicine, Lucian Blaga University of Sibiu, 550024 Sibiu, Romania; liana.chicea@ulbsibiu.ro; 3 Internal Medicine Department, Academic Emergency Hospital, 550245 Sibiu, Romania; 4 Department of Allergology and Immunology, “Iuliu Hatieganu” University of Medicine and Pharmacy, 400012 Cluj-Napoca, Romania; irena.nedelea@umfcluj.ro (I.N.); diana.deleanu@umfcluj.ro (D.D.); 5 Bioclinica Medical Analysis Laboratory, 310300 Arad, Romania; carmenstrauti@gmail.com; 6 Department of General Medicine, “Vasile Goldis” Western University of Arad, Blvd. Revolutiei, No. 96, 310025 Arad, Romania; 7 Clinical Hematology Department, “Vasile Goldis” Western University of Arad, 310025 Arad, Romania; 8 Center for Research and Innovation in Precision Medicine of Respiratory Diseases, “Victor Babes” University of Medicine and Pharmacy, Eftimie Murgu Square 2, 300041 Timisoara, Romania; oancea@umft.ro

**Keywords:** non-Hodgkin lymphoma, urinary tract infections, immunodeficiency, uropathogens, COVID-19, severe, SARS-CoV-2

## Abstract

**Background:** Non-Hodgkin lymphoma (NHL) is a malignancy of the immune system that includes several subtypes, most commonly diffuse large B-cell lymphoma and follicular lymphoma. Its etiology is multifactorial, with risk factors such as immunosuppressive therapy, infections, chemical exposure, and advanced age. A key aspect is the bidirectional relationship between lymphoma and immunodeficiency, which increases susceptibility to recurrent infections and complicates disease management. **Case presentation:** One particularly challenging case during the COVID-19 pandemic involved a patient with a personal history of diffuse B-cell non-Hodgkin lymphoma, diagnosed 5 years earlier, who had undergone eight cycles of rituximab-based chemotherapy. The patient tested positive for SARS-CoV-2 for three consecutive months and experienced repeated urinary tract infections warranting more in-depth investigations. The uniqueness of this case lies in the rare association of non-Hodgkin lymphoma, suspected post-rituximab immunodeficiency, severe COVID-19 infection, and recurrent urinary tract infections, which complicated clinical management. Despite appropriate treatment for both respiratory and urinary infections, as well as eight cycles of chemotherapy, the patient’s condition continued to deteriorate significantly, ultimately requiring intravenous immunoglobulin replacement therapy. Following the treatment, the patient presented a remarkable clinical improvement, with resolution of the signs and symptoms, and an absence of further recurrent infections. The patient remained clinically stable under regular immunoglobulin replacement therapy, with sustained infection control and improved quality of life. **Conclusions:** This case highlights the importance of assessing immune status in patients with a hematological malignancy, especially when recurrent infections persist.

## 1. Introduction

Multidrug-resistant urinary tract infections (MDR UTIs) have become a major contributor to hospital-acquired infections worldwide. According to recent epidemiological data, pathogens such as carbapenem-resistant Enterobacterales (CRE) and *Pseudomonas aeruginosa* are increasingly detected in clinical settings and can exhibit resistance even to last-resort agents, including colistin and ceftazidime/avibactam [1]. Mechanistic studies indicate that biofilm formation, horizontal gene transfer, and carbapenemase production further limit therapeutic options and are associated with longer hospital stays and higher mortality. These challenges are particularly severe in immunocompromised patients, for whom infection control and antimicrobial stewardship are essential components of care [2].

Patients with hematological malignancies, especially those treated with B-cell–depleting agents such as rituximab, are highly vulnerable to severe and persistent infections because of both quantitative and qualitative immune defects. In addition to B-cell depletion, cytokine imbalance, impaired antigen presentation, and T-cell dysregulation further compromise immune defense. Hypogammaglobulinemia following rituximab therapy may persist for months or years, predisposing patients to recurrent bacterial and viral infections and diminishing vaccine responsiveness [3,4,5].

The COVID-19 pandemic has added further complexity to this scenario. Persistent SARS-CoV-2 infection has been documented in patients with secondary immunodeficiency, often accompanied by co-infection with multidrug-resistant bacteria and poor immune recovery [6,7]. This case illustrates the convergence of rituximab-induced hypogammaglobulinemia, recurrent MDR urinary tract infections, and prolonged SARS-CoV-2 infection. It underscores the importance of early immune assessment, timely immunoglobulin replacement, and coordinated multidisciplinary management. Beyond its clinical relevance, the case highlights a critical overlap between oncology, infectious diseases, and immunology and points to the need for predictive biomarkers to guide personalized interventions in immunocompromised populations [8,9].

## 2. Case Study

At the initial presentation, a 42-year-old patient, with a prior history of diffuse B-cell non-Hodgkin lymphoma, type 2 diabetes, dyslipidemia, and hepatic steatosis, presented high-grade fever (39.5 °C), pronounced fatigue, asthenia, persistent dry cough, and exertional dyspnea, corresponding to modified Medical Research Council (mMRC) grade 2. Laboratory tests and imaging were performed to assess the severity of COVID-19 infection and potential complications. Arterial blood gas (ABG) analysis revealed mild hypoxemia, with a pH of 7.45, PaO_2_ of 58 mmHg, PaCO_2_ of 36 mmHg, bicarbonate (HCO_3_^−^) of 24 mEq/L, and oxygen saturation (SaO_2_) of 89%.

**Figure 1:**
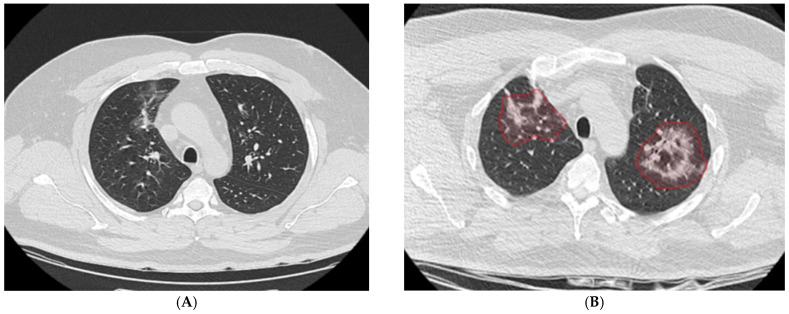
Progressive pulmonary changes on chest CT imaging in a 45-year-old patient with diffuse large *B-cell non-Hodgkin lymphoma*. (**A**) Baseline chest CT scan showing discrete bilateral ground-glass opacities and mild peribronchovascular thickening, with limited parenchymal involvement. (**B**) Follow-up chest CT scan demonstrating marked progression to bilateral consolidations with central ground-glass opacities and peripheral reticulation, highlighted in red, predominantly affecting the upper lobes.

Initial chest computed tomography (CT) demonstrated discrete bilateral ground-glass opacities and peribronchovascular thickening with limited parenchymal extension. During a subsequent hospitalization, follow-up CT imaging showed progression, with bilateral areas of consolidation containing central ground-glass components and peripheral reticulation, predominantly in the upper lobes. These findings suggested an evolving fibrotic process despite ongoing therapy (Figure 1A). Panel B illustrates marked progression to bilateral consolidations with central ground-glass opacities and peripheral reticulation, highlighted in red, predominantly involving the upper lobes (Figure 1B).

Laboratory investigations at initial presentation revealed a marked inflammatory response and hematologic abnormalities. Elevated levels of interleukin-6 (IL-6), ferritin, C-reactive protein (CRP), fibrinogen, and lactate dehydrogenase (LDH) were consistent with a hyperinflammatory state. In contrast, leukopenia and lymphopenia were evident, indicating immune dysregulation. These findings are summarized in Table 1.

After the initial screening the patient received a treatment regimen comprising corticosteroids, anticoagulants, intravenous fluids and electrolytes, and supportive care. Following hospitalization, clinical improvement was observed, with normalization of most laboratory parameters except for persistently elevated ferritin levels. Given the stable clinical condition, resolution of most signs and symptoms, and absence of hypoxemia or other complications, the patient was discharged after 12 days of hospitalization. Close outpatient monitoring was arranged to ensure safety and follow-up of laboratory parameters and clinical status.

Two weeks post-discharge, the patient experienced a relapse with high-grade fever (39 °C), fatigue, and exertional dyspnea, prompting readmission. Another SARS-CoV-2 RT-PCR test was performed and was again positive (Table 2).

These findings, combined with the patient’s history of rituximab-based chemotherapy received until 2018, following a 2017 diagnosis of NHL confirmed by an excisional lymph node biopsy when the disease entered remission, raised suspicion of rituximab-induced B-cell depletion contributing to persistent infection and immune dysfunction.

The recurrence of clinical signs, in the context of persistently positive RT-PCR tests for SARS-CoV-2, raised concern for impaired viral clearance. The patient’s prior treatment with rituximab, an anti-CD20 monoclonal antibody, was considered a contributing factor, given its association with prolonged B-cell depletion and impaired humoral immunity; notably, the last dose had been administered in January 2022.

Peripheral blood smear revealed lymphopenia with predominantly small mature lymphocytes, occasional atypical lymphocytes, and no significant dysplasia. Neutrophils presented normal morphology. Bone marrow aspiration and biopsy demonstrated normocellular marrow without evidence of malignant infiltration. Myeloid and erythroid lineages were preserved. These findings corroborated the laboratory evidence of impaired humoral immunity and persistent cytopenias.

The patient experienced recurrent UTIs throughout his life, both during childhood and adulthood, despite urine culture testing, bacterial identification, and targeted antibiotic therapy according to antibiogram results, as well as adherence to strict hygiene measures. During the year of observation, several episodes of UTIs occurred, often coinciding with SARS-CoV-2 exacerbations, suggesting a possible interplay between bacterial infections and virus-induced immune dysregulation. These episodes may have contributed to systemic inflammation and clinical deterioration, further complicating the patient’s management.

Urinalysis performed during multiple hospitalizations revealed leukocyturia (10–15 WBC/hpf), occasional hematuria (1–2 RBC/hpf), and mild proteinuria. Nitrites were intermittently positive, consistent with Gram-negative bacteriuria. Some episodes of bacteriuria were asymptomatic, highlighting the occurrence of asymptomatic bacteriuria, especially during periods of immunosuppression. Recurrent urinary tract infections (UTIs) were often associated with mild dysuria, urinary frequency, urgency, and suprapubic discomfort. No flank pain or signs of pyelonephritis were observed in most episodes.

Urine cultures repeatedly identified Gram-negative pathogens, initially including *Escherichia coli*, *Klebsiella pneumoniae*, and *Proteus* spp., with subsequent isolations of *Pseudomonas aeruginosa* during later hospitalizations, reflecting a shift toward opportunistic and nosocomial pathogens. Antimicrobial susceptibility testing revealed extensive multidrug resistance, with *K. pneumoniae* resistant to all tested classes except imipenem and *P. aeruginosa* sensitive only to meropenem. These findings, summarized in Table 3, underscored the emergence of multidrug-resistant organisms that severely restricted therapeutic options and necessitated parenteral use of reserve carbapenems under close monitoring. In October 2022, *Proteus mirabilis* was cultured, resistant to all classes except carbapenems, requiring targeted therapy with meropenem.

Over the last ten years, the patient experienced multiple episodes of culture-confirmed urinary tract infections, initially caused by Escherichia coli and later by multidrug-resistant strains (Klebsiella pneumoniae, Proteus mirabilis, and Pseudomonas aeruginosa).

Between 2013 and 2018, the infections were sporadic and responsive to oral fluoroquinolones (ciprofloxacin, levofloxacin) or beta-lactams (amoxicillin/clavulanate, cefuroxime).

From 2019 to 2021, treatment courses included ceftriaxone, ceftazidime, or trimethoprim-sulfamethoxazole, with partial responses and recurrent infections.

In the last two years (2022–2023), multidrug-resistant organisms emerged. The patient required parenteral carbapenems (imipenem or meropenem) following identification of extended-spectrum beta-lactamase (ESBL)-producing *Klebsiella pneumoniae* and *Proteus mirabilis*.

Urine samples were cultured on chromID CPS Elite medium (bioMérieux), and pathogen identification and antimicrobial susceptibility testing were performed using the Vitek 2 Compact system (bioMérieux) according to the EUCAST (2025) guidelines [1]. Multidrug-resistant (MDR) and extensively drug-resistant (XDR) phenotypes were defined following the ECDC/CDC joint criteria. MDR was defined as non-susceptibility to at least one agent in three or more antimicrobial categories, whereas XDR denoted non-susceptibility to all but two or fewer antimicrobial classes [2].

Antibiotic therapy was selected based on AST profiles, with reserve carbapenems (imipenem or meropenem) administered only after resistance to all other tested agents was confirmed.

The progressive shift from *Escherichia coli* to *Klebsiella pneumoniae* and *Pseudomonas aeruginosa* over the course of hospitalization reflected a transition from community-acquired to opportunistic, healthcare-associated pathogens. Abdominal ultrasonography and systemic evaluation excluded alternative infectious foci, supporting the urinary tract as the primary site of infection.

For the purpose of this study, extensively drug-resistant (XDR) and multidrug-resistant (MDR) phenotypes were classified according to the ECDC/CDC consensus definitions [2]. Accordingly, MDR was defined as non-susceptibility to at least one agent in ≥3 antimicrobial categories, and XDR as non-susceptibility to all but ≤2 categories. These criteria were applied to the susceptibility profiles generated by the Vitek 2 Compact (bioMérieux) system, interpreted following EUCAST 2025 guidelines [1].

**Figure 2:**
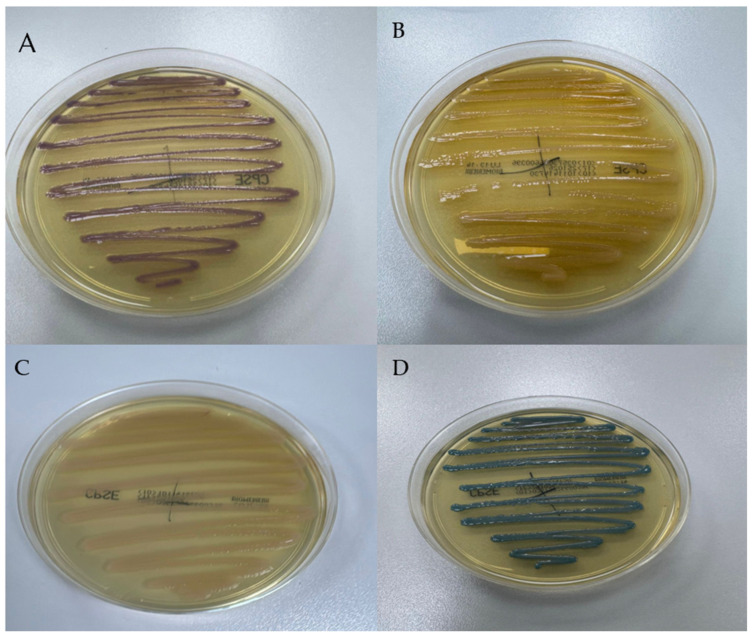
Colony morphology of uropathogens isolated from the urine of a 42-year-old male patient with non-Hodgkin large B-cell lymphoma, suspected primary immunodeficiency, and recurrent urinary tract infections, cultured on chromID CPS Elite medium (bioMérieux). Species identification was confirmed using Vitek 2 (bioMérieux): (**A**) Escherichia coli showing pink-to-burgundy colonies; (**B**) Proteus mirabilis producing light-beige colonies; (**C**) Pseudomonas aeruginosa with yellowish colonies; (**D**) Klebsiella/Enterobacter/Serratia group presenting green-to-blue colonies. Abdominal ultrasonography was performed during hospitalization in the nephrology ward, demonstrating normal renal size and morphology, with no evidence of hydronephrosis, lithiasis, or structural abnormalities of the urinary tract. The bladder wall appeared regular, with no post-void residual volume or mucosal thickening, effectively excluding obstructive or anatomic causes for recurrent infections. A contrast-enhanced CT scan of the abdomen and pelvis confirmed the absence of congenital or structural urinary tract anomalies. The patient had no history of voiding dysfunction or neurogenic bladder. Urodynamic studies and cystoscopy were therefore not indicated.

The patient experienced recurrent urinary tract infections caused by multiple multidrug-resistant (MDR) uropathogens over a four-month period, as summarized in Table 3. To further characterize these isolates, urine cultures were analyzed, and their colony morphology was documented, as illustrated in Figure 2.

The management of recurrent UTIs caused by XDR organisms posed additional challenges. Urine cultures grew Klebsiella pneumoniae and Pseudomonas aeruginosa with limited antimicrobial susceptibility profiles, necessitating the use of broad-spectrum, reserve antibiotics such as imipenem and meropenem for effective treatment. Antibiotic prophylaxis was not prescribed because of the high risk of further resistance selection and limited oral options due to the MDR profile. Current guidelines recommend avoiding routine prophylaxis in such cases, focusing instead on targeted therapy and immunoglobulin replacement [10,11].

As part of the evaluation for recurrent infections and protracted SARS-CoV-2 positivity, comprehensive immunological testing was performed. Serum protein electrophoresis revealed a reduced gamma-globulin fraction (4.6%, reference range: 11.1–18.8%) and an elevated albumin-to-globulin ratio (A/G) ratio of 2.3 (reference range: 1.0–1.5). These findings were suggestive of hypogammaglobulinemia.

Further immunophenotyping demonstrated a complete absence of CD19+ B lymphocytes, consistent with profound B-cell depletion, most likely secondary to prior rituximab therapy for NHL. Quantification of immunoglobulins revealed marked reductions in all major classes: IgG at 321 mg/dL (reference: 700–1600 mg/dL), IgA at 29 mg/dL (reference: 70–400 mg/dL), and IgM at 9.3 mg/dL (reference: 40–230 mg/dL). Complement fractions were mildly elevated, with C3 at 187 mg/dL (reference: 90–180 mg/dL) and C4 at 43 mg/dL (reference: 10–40 mg/dL).

Given the patient’s immunocompromised state, comprehensive viral screening was performed, including HIV, hepatitis B and C, cytomegalovirus (CMV), and Epstein–Barr virus (EBV), all of which returned negative.

**Figure 3:**
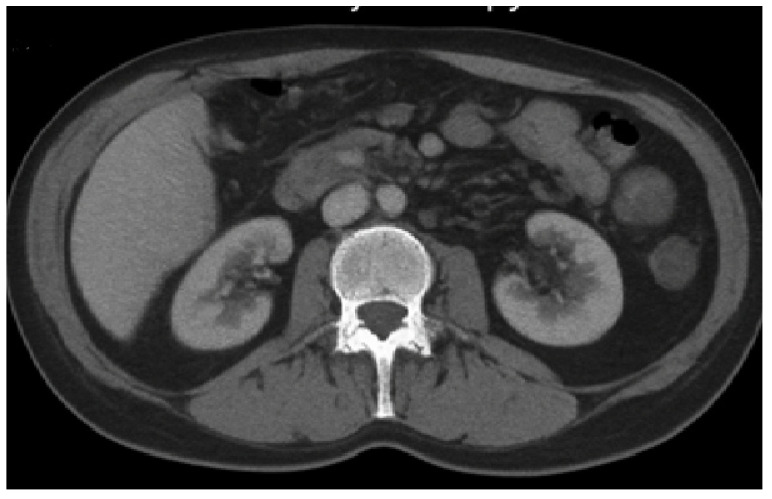
A contrast-enhanced CT scan of the abdomen and pelvis.

**Figure 4:**
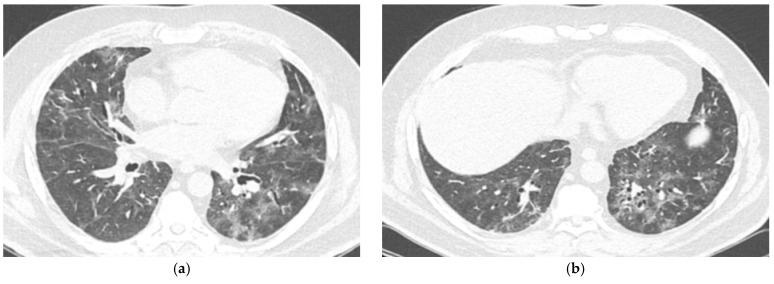
Rapidly advancing pulmonary involvement on chest CT imaging in an 45 year old patient. (**a**) Chest CT scan showing bilateral diffuse ground-glass opacities in the lower lobes. (**b**) Chest CT scan demonstrating progression with extensive ground-glass opacities and interlobular septal thickening, involving approximately 50% of the pulmonary parenchyma.

Chest CT imaging (Figure 3 and Figure 4), performed approximately one month after the initial CT, demonstrated diffuse ground-glass opacities affecting about 50% of the pulmonary parenchyma, consistent with rapidly progressive pulmonary involvement.

Immunophenotyping of peripheral blood lymphocytes revealed a mild leukocytosis with discrete alterations in T cell subpopulations. The profile showed reduced naïve T lymphocytes (CD45RA+) and a relative predominance of memory T lymphocytes (CD45RO+), alongside a moderate increase in activated T cells (HLA-DR+). In addition, the CD4/CD8 ratio was found to be inverted, suggesting ongoing immune activation. No circulating B lymphocytes were detected, and serum protein electrophoresis excluded the presence of monoclonal bands. These findings collectively indicate a dysregulated immune response, which may affect susceptibility to infections and responsiveness to antimicrobial therapy.

Following the identification of hypogammaglobulinemia, the patient was started on intravenous immunoglobulin (IVIG) therapy with Intratect, followed by subcutaneous immunoglobulin replacement therapy (HyQvia) to ensure long-term immune protection. This intervention resulted in clinical stabilization, resolution of fever, and normalization of inflammatory markers. At this stage, inflammatory parameters showed no significant elevation, with CRP at 1.77 mg/L (reference: 0–5 mg/L) and rheumatoid factor (RF) < 6.08 IU/mL (reference: 0–14 IU/mL).

The constellation of hypogammaglobulinemia, absence of B cells, and recurrent infections supported the diagnosis of secondary humoral immunodeficiency, with features overlapping those of common variable immunodeficiency (CVID). Genetic testing for primary immunodeficiency was not performed, as the clinical and immunological findings were attributed to secondary B-cell depletion following rituximab therapy.

These findings confirmed the presence of a severe combined immunoglobulin deficiency in the setting of B-cell depletion. The immunodeficiency was considered secondary to anti-CD20 monoclonal antibody therapy and was thought to underlie the patient’s susceptibility to recurrent UTIs and protracted SARS-CoV-2 infection.

On 25 September 2022, the patient received Intratect (human immunoglobulin) with a marked clinical and biological response, including normalization of IL-6 and CRP levels. Immunophenotyping, performed as before, revealed profound B-cell depletion (absence of CD19+ lymphocytes), confirming a persistent secondary humoral immunodeficiency.

To ensure sustained immune protection, the patient was transitioned to long-term subcutaneous immunoglobulin replacement therapy (HyQvia). This therapeutic strategy resulted in resolution of fever, improvement in energy levels, and prevention of further severe infections during outpatient follow-up (Table 4 and Table 5).

Following comprehensive clinical and paraclinical investigations, the following diagnoses were established: bronchopneumonia associated with SARS-CoV-2 infection, CVID, type 2 diabetes mellitus treated with oral antidiabetic drugs, mixed dyslipidemia (total cholesterol: 240 mg/dL, LDL-cholesterol: 170 mg/dL, HDL-cholesterol: 35 mg/dL, triglycerides: 220 mg/dL), hepatic steatosis (confirmed by abdominal ultrasound showing increased echogenicity of the liver consistent with fatty infiltration), and NHL.

Long-term management involved multidisciplinary care, with regular immunological monitoring.

**Figure 5:**
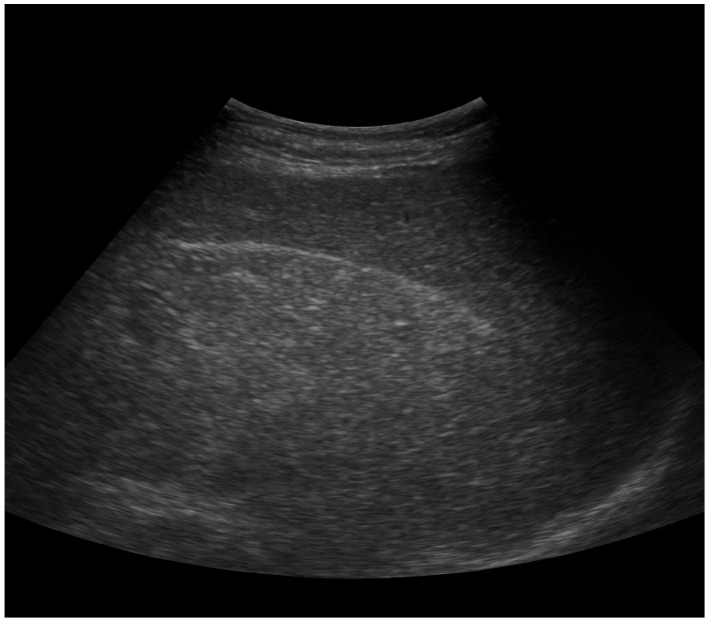
Abdominal ultrasound showing increased liver echogenicity consistent with hepatic steatosis (fatty infiltration).

The patient’s urinary tract infections were consistent with lower urinary tract infection (cystitis). He presented with dysuria, urinary frequency, urgency, and suprapubic discomfort, but no flank pain, costovertebral angle tenderness, or systemic features of pyelonephritis were observed. Abdominal ultrasonography demonstrated normal renal morphology and excluded hydronephrosis or parenchymal involvement, confirming the absence of upper urinary tract infection (Figure 5).

## 3. Discussion

UTIs are a common complication in immunocompromised individuals, and their management is significantly complicated by the emergence of MDR organisms. In this patient, recurrent UTIs were caused predominantly by *Escherichia coli*, *Klebsiella pneumoniae*, and later *Pseudomonas aeruginosa*, pathogens increasingly recognized for their ability to acquire resistance determinants in both community and healthcare settings [9]. Notably, *Klebsiella pneumoniae* exhibited an XDR phenotype, resistant to all tested antibiotic classes except carbapenems, colistin, and ceftazidime/avibactam [12,13]. Similarly, *Pseudomonas aeruginosa* demonstrated resistance to beta-lactams, fluoroquinolones, and aminoglycosides, retaining susceptibility only to meropenem, colistin, and ceftazidime/avibactam. This pattern underscores the limited therapeutic options and highlights the importance of antimicrobial stewardship and close microbiological monitoring [14].

The recurrent need for prolonged carbapenem courses emphasizes the scarcity of effective treatments and the potential role of alternative strategies, such as combination therapy, novel beta-lactamase inhibitors, and bacteriophage therapy, though their efficacy in severely immunosuppressed patients remains uncertain [15]. The clinical course of this patient highlights a complex bidirectional relationship between severe SARS-CoV-2 infection and recurrent UTIs, which often coincided and may have exacerbated systemic inflammation and immune dysregulation. COVID-19 is known to induce profound alterations in both innate and adaptive immunity, including lymphopenia, cytokine dysregulation, and transient impairment of mucosal barriers, increasing susceptibility to secondary bacterial infections [16,17].

SARS-CoV-2 infection can compromise mucosal immunity through multiple mechanisms. The virus utilizes the ACE2 receptor, expressed not only in pulmonary but also in urothelial epithelial cells, leading to local epithelial injury and impaired barrier integrity. This disruption facilitates bacterial adherence and invasion. In addition, COVID-19 is associated with lymphopenia, reduced mucosal dendritic cell activity, and decreased secretion of antimicrobial peptides and secretory IgA, which are essential for maintaining urinary tract sterility. The cytokine imbalance (particularly elevated IL-6 and TNF-α) further inhibits IgA class switching and impairs epithelial regeneration. In immunocompromised individuals with rituximab-induced B-cell depletion, these effects are magnified, resulting in profound mucosal immune dysfunction and increased vulnerability to recurrent bacterial infections. Persistent mucosal inflammation and impaired bacterial clearance can progress to urosepsis, particularly when multidrug-resistant Gram-negative pathogens are involved.

Episodes of UTI due to MDR Gram-negative pathogens frequently overlapped with SARS-CoV-2 relapses, suggesting a synergistic effect. Bacterial infections may have acted as additional pro-inflammatory stimuli, perpetuating cytokine-driven systemic inflammation already observed in severe COVID-19 [18]. Conversely, recurrent bacteriuria and episodes of urosepsis could have contributed to immune exhaustion and impaired viral clearance. Urosepsis was defined according to Sepsis-3 criteria as a suspected urinary tract infection associated with a rise in the Sequential Organ Failure Assessment (SOFA) score of ≥2 points, indicating organ dysfunction [19]. This vicious cycle likely fueled the patient’s persistent fever, respiratory deterioration, and prolonged PCR positivity for SARS-CoV-2 [20].

The presence of leukocyturia, combined with positive cultures, underscores ongoing urinary tract inflammation even in the absence of overt symptoms. Serial SARS-CoV-2 IgG antibody testing failed to demonstrate seroconversion, supporting an impaired humoral response, while T-cell enumeration and functional assays suggested preserved cellular immunity [21]. The markedly elevated ferritin and IL-6 levels, combined with persistent lymphopenia, were suggestive of sustained cytokine-driven inflammation on a background of incomplete viral clearance, with favorable response to corticosteroid therapy further supporting an inflammatory-dominant mechanism [22].

The co-occurrence of infections required careful coordination of antimicrobial and immunomodulatory therapies. While corticosteroids controlled hyper-inflammation, they also carried a risk of transient immunosuppression and bacterial superinfection, highlighting the need to balance anti-inflammatory treatment with vigilant microbiological surveillance [23]. Multidisciplinary care, including infectious disease specialists, immunologists, and microbiologists, was pivotal in optimizing outcomes. Stringent infection control measures and targeted prophylaxis should be considered to prevent nosocomial colonization and reduce MDR organism burden [10].

The immunological profile, characterized by profound hypogammaglobulinemia, absence of CD19+ B lymphocytes, and recurrent infections, raised the question of distinguishing between primary and secondary immunodeficiency [11]. While CVID is diagnosed based on reduced IgG, IgA, and/or IgM with impaired antibody responses, the temporal relationship with rituximab therapy strongly suggests a secondary humoral immune defect. Anti-CD20 therapy is known to induce prolonged B-cell depletion, predisposing to opportunistic infections [24]. Serial SARS-CoV-2 antibody testing failed to demonstrate seroconversion despite vaccination and infection, confirming a profound deficit in humoral responses attributable to B-cell depletion [25].

Nevertheless, the patient’s history of recurrent infections since childhood raises the possibility of an underlying primary immunodeficiency, potentially unmasked or exacerbated by immunosuppressive therapy. Late-onset CVID or previously unrecognized antibody deficiencies can manifest with recurrent infections even before immunosuppressive therapy [26]. Although genetic testing was not performed, the strong temporal association between rituximab and B-cell depletion favors a secondary etiology. Distinguishing primary from secondary immunodeficiency is clinically relevant due to differences in prognosis, monitoring, and family counseling, though in both cases immunoglobulin replacement remains a cornerstone of management [27,28].

Immunoglobulin replacement therapy, initiated with IVIG and transitioned to subcutaneous HyQvia, resulted in resolution of fever, stabilization of inflammatory markers, and prevention of severe infections. The prolonged need for carbapenem therapy underscores the importance of antimicrobial stewardship to minimize the emergence of CRE and MDR Pseudomonas [28]. Co-administration of corticosteroids during SARS-CoV-2 exacerbations required individualized assessment, balancing anti-inflammatory benefit with infection risk [29].

Finally, optimizing vaccination timing in patients receiving B-cell-depleting therapies remains challenging, with strategies such as pre-rituximab vaccination or post-therapy revaccination under investigation to improve protective immunity [30]. Despite profound immunodeficiency, the patient’s partial innate immune responses, evidenced by leukocyturia and dysuria, suggest some preserved functionality [31,32].

The clinical presentation of dysuria, urinary frequency, urgency, and suprapubic discomfort, together with laboratory findings of leukocyturia, bacteriuria, and mild proteinuria, in the absence of fever or upper tract involvement, supports the diagnosis of recurrent lower urinary tract infections (cystitis) in our patient.

Being a single-patient case report, this study has inherent limitations regarding generalizability [33,34]. Lack of genetic testing and serial cytokine profiling restricts definitive conclusions on primary versus secondary immunodeficiency and host–virus interactions [33]. Nonetheless, detailed longitudinal microbiological and immunological data provide valuable insights into diagnostic and therapeutic challenges in managing immunocompromised patients, highlighting the need for further studies with larger cohorts and advanced immunogenetic analyses [35,36]. The main limitation of this report is the lack of baseline immunological data prior to rituximab administration, which makes it impossible to definitively distinguish between pre-existing and therapy-induced immune dysfunction. Additionally, as the patient was managed in a non-specialized center, systematic immune monitoring was not routinely performed, and the identification of this case was incidental. Therefore, the findings should be interpreted with caution, and larger studies from immunology-focused centers are needed to better characterize similar cases.

## 4. Clinical Take-Home Messages

Recurrent UTIs in immunocompromised patients warrant detailed immune assessment.

Exclude structural causes (ultrasound, CT, cystoscopy if indicated).

Perform extended AST using EUCAST standards to identify MDR/XDR.

Consider secondary immunodeficiency after B-cell–depleting therapies.

Early IVIG or SCIG replacement can stabilize immune function and prevent recurrence.

Regular monitoring of serum immunoglobulin levels is recommended before and at intervals during and after rituximab therapy, especially in patients with recurrent or severe infections.

## 5. Conclusions

This case underscores the complex interplay between primary humoral immunodeficiency, persistent SARS-CoV-2 infection, and MDR bacterial pathogens in an immunocompromised host. Rituximab-induced B-cell depletion led to profound hypogammaglobulinemia, impaired viral clearance, and recurrent UTIs caused by XDR Klebsiella pneumoniae and Pseudomonas aeruginosa. These infections required prolonged carbapenem therapy, highlighting the therapeutic challenges posed by antimicrobial resistance in vulnerable populations. Early recognition of immunodeficiency, implementation of immunoglobulin replacement therapy, and strict antimicrobial stewardship were critical in achieving clinical stabilization and preventing further infectious complications.

## Figures and Tables

**Table 1:** Laboratory parameters at initial presentation.

Parameter	Patient Value	Reference Range (Normal)
Interleukin-6 (IL-6)	27 pg/mL	<7 pg/mL
Ferritin	1173 ng/mL	30–400 ng/mL (men)
C-reactive protein (CRP)	85 mg/L	<5 mg/L
Fibrinogen	687 mg/dL	200–400 mg/dL
Lactate dehydrogenase (LDH)	412 U/L	140–280 U/L
Leukocytes (WBC)	2.77 × 10^9^/L	4–10 × 10^9^/L
Lymphocytes (relative)	12.4%	20–40%

**Table 2:** Laboratory parameters at readmission of the 45-year-old patient, two weeks after the initial admission.

Parameter	Patient Value	Reference Range (Normal)
Ferritin	2000 ng/mL	30–400 ng/mL (men)
Interleukin-6 (IL-6)	89 pg/mL	<7 pg/mL
C-reactive protein (CRP)	87 mg/L	<5 mg/L
Leukocytes (WBC)	2.14 × 10^9^/L	4–10 × 10^9^/L

**Table 3:** Timeline of uropathogens and their antimicrobial susceptibility patterns.

Date	Pathogen	Resistant to	Sensitive to
June 2022	*Escherichia coli*	Amoxicillin/clavulanate, Ciprofloxacin, Gentamicin, Temocillin	Imipenem, Meropenem
August 2022	*Klebsiella pneumoniae*	Amoxicillin/clavulanate, Ceftriaxone, Ceftazidime, Cefepime, Ciprofloxacin, Levofloxacin, Gentamicin, Amikacin, Tobramycin, Colistin, Temocillin	Imipenem, Meropenem
September 2022	*Pseudomonas aeruginosa*	Piperacillin/tazobactam, Ceftazidime, Cefepime, Ciprofloxacin, Levofloxacin, Gentamicin, Amikacin, Tobramycin	Meropenem
October 2022	*Proteus mirabilis*	Amoxicillin/clavulanate, Ceftriaxone, Cefepime, Ciprofloxacin, Levofloxacin, Gentamicin, Amikacin, Tobramycin, Colistin, Temocillin	Meropenem

**Table 4:** Inpatient treatment at secondary presentation.

Medication/Therapy	Dose/Route	Duration/Timing	Purpose
Human immunoglobulin (Panzyga)	IV	Two infusions	Correction of hypogammaglobulinemia
Corticosteroids (dexamethasone)	8 mg IV	During hospitalization	Anti-inflammatory, immunomodulation
Anticoagulants (rivaroxaban)	10 mg orally	As indicated	Thromboprophylaxis
Intravenous fluids and electrolytes	-	As needed	Supportive care
Mucolytic agents	-	During hospitalization	Respiratory support
Nutritional immune support (Fort Imuno)	-	During hospitalization	Support immune function
Antidiabetic therapy	-	Continuous	Glycemic control
Arginine supplementation	-	As indicated	Nutritional support
Proton pump inhibitor (esomeprazole)	20 mg orally	During hospitalization	Gastroprotection

**Table 5:** Outpatient treatment at discharge.

Medication/Therapy	Dose/Route	Purpose
Dexamethasone	8 mg orally daily	Anti-inflammatory
Rivaroxaban	10 mg orally daily	Thromboprophylaxis
Antidiabetic therapy	As per prior regimen	Glycemic control
Fort Imuno	As per manufacturer	Nutritional immune support
Arginine supplementation	As indicated	Nutritional support
Esomeprazole	20 mg orally daily	Gastroprotection

## Data Availability

Data are contained within the article.

## Abbreviations

The following abbreviations are used in this manuscript:

ABG	Arterial Blood Gas
A/G	Albumin-to-Globulin ratio
CLSI	Clinical and Laboratory Standards Institute
COVID-19	Coronavirus Disease 2019
CRE	Carbapenem-Resistant Enterobacterales
CRP	C-Reactive Protein
CVID	Common Variable Immunodeficiency
CT	Computed Tomography
EUCAST	European Committee on Antimicrobial Susceptibility Testing
HCO_3_^−^	Bicarbonate
IVIG	Intravenous Immunoglobulin
LDH	Lactate Dehydrogenase
MDR	Multidrug-Resistant
mMRC	Modified Medical Research Council
NHL	Non-Hodgkin Lymphoma
PaCO_2_	Partial pressure of Carbon Dioxide in Arterial Blood
PaO_2_	Partial Pressure of Oxygen in Arterial Blood
PID	Primary Immunodeficiency
RF	Rheumatoid Factor
RT-PCR	Reverse Transcription Polymerase Chain Reaction
SaO_2_	Oxygen Saturation
SID	Secondary Immunodeficiency
SARS-CoV-2	Severe Acute Respiratory Syndrome Coronavirus 2
UTI	Urinary Tract Infection
XDR	Extensively Drug-Resistant
